# Clinical and Genomic Epidemiology of Coxsackievirus A21 and Enterovirus D68 in Homeless Shelters, King County, Washington, USA, 2019–2021

**DOI:** 10.3201/eid3011.240687

**Published:** 2024-11

**Authors:** Sarah N. Cox, Amanda M. Casto, Nicholas M. Franko, Eric J. Chow, Peter D. Han, Luis Gamboa, Brian Pfau, Hong Xie, Kevin Kong, Jaydee Sereewit, Melissa A. Rolfes, Emily Mosites, Timothy M. Uyeki, Alexander L. Greninger, Marco Carone, M. Mia Shim, Trevor Bedford, Jay Shendure, Michael Boeckh, Janet A. Englund, Lea M. Starita, Pavitra Roychoudhury, Helen Y. Chu

**Affiliations:** University of Washington, Seattle, Washington, USA (S.N. Cox, A.M. Casto, N.M. Franko, E.J. Chow, H. Xie, K. Kong, J. Sereewit, A.L. Greninger, M.M. Shim, T. Bedford, J. Shendure, M. Boeckh, J.A. Englund, L.M. Starita, P. Roychoudhury, H.Y. Chu); Public Health Seattle and King County, Seattle (E.J. Chow, M.M. Shim); Brotman Baty Institute for Precision Medicine, Seattle (P.D. Han, L. Gamboa, B. Pfau, T. Bedford, J. Shendure, L.M. Starita); Centers for Disease Control and Prevention, Atlanta, Georgia, USA (M.A. Rolfes, E. Mosites, T.M. Uyeki); Fred Hutchinson Cancer Research Center, Seattle (A.L. Greninger, M. Carone, T. Bedford, M. Boeckh, P. Roychoudhury); Howard Hughes Medical Institute, Seattle (T. Bedford, J. Shendure); Seattle Children’s Research Institute, Seattle (J.A. Englund)

**Keywords:** enterovirus, coxsackievirus, viruses, enterovirus C, human, enterovirus D, human, enterovirus A, human, ill-housed persons, homeless, congregate settings, communicable diseases, disease outbreaks, cross-sectional studies, genome, viral, King County, Washington, United States

## Abstract

Congregate homeless shelters are disproportionately affected by infectious disease outbreaks. We describe enterovirus epidemiology across 23 adult and family shelters in King County, Washington, USA, during October 2019–May 2021, by using repeated cross-sectional respiratory illness and environmental surveillance and viral genome sequencing. Among 3,281 participants >3 months of age, we identified coxsackievirus A21 (CVA21) in 39 adult residents (3.0% [95% CI 1.9%–4.8%] detection) across 7 shelters during October 2019–February 2020. We identified enterovirus D68 (EV-D68) in 5 adult residents in 2 shelters during October–November 2019. Of 812 environmental samples, 1 was EV-D68–positive and 5 were CVA21–positive. Other enteroviruses detected among residents, but not in environmental samples, included coxsackievirus A6/A4 in 3 children. No enteroviruses were detected during April 2020–May 2021. Phylogenetically clustered CVA21 and EV-D68 cases occurred in some shelters. Some shelters also hosted multiple CVA21 lineages.

Enteroviruses are responsible for ≈10–15 million symptomatic illnesses in the United States annually; however, epidemiologic surveillance and genetic characterization of many enterovirus subspecies is limited (*1*–*3*). Coxsackievirus A21 (CVA21), discovered in 1947, and enterovirus D68 (EV-D68), discovered in 1962, can cause illnesses ranging from cold-like symptoms to difficulty breathing and wheezing (*2*,*4*,*5*–*9*). In recent years, interest and awareness of EV-D68 has grown because of temporal and geographic associations of outbreaks with clusters of acute flaccid myelitis in children (*4*,*5*). No specific treatments or vaccines are available for nonpolio enteroviruses (*4*), and the pathogenesis of the infections remain poorly understood (*10*). A need exists for phylogeographic epidemiology to define genomic variation and genetic changes over time and to determine transmission patterns in the community (*5*,*11*,*12*).

Persons experiencing homelessness are at increased risk for infectious diseases and complications, such as influenza, COVID-19, and hepatitis A (*13*,*14*). The risk for acquiring infections is considerably higher for those who live in congregate shelters because of challenges with overcrowding, maintaining physical distance, poor ventilation, and sharing of hygiene facilities (*15*–*18*). To our knowledge, minimal data are available to describe enterovirus transmission among persons experiencing homelessness.

Our study aimed to characterize the epidemiology of nonrhinovirus enteroviruses through nasal swab specimens and environmental samples collected from homeless shelters across King County, Washington, USA, during 2019–2021. We used genomic sequencing to describe the molecular diversity of enteroviruses within and across shelter sites.

## Materials and Methods

### Study Design and Population

We retrospectively analyzed cross-sectional respiratory virus surveillance data collected during October 1, 2019–May 31, 2021, across 23 homeless shelters in King County, which includes the city of Seattle. As previously described, the Seattle Flu Study instituted active routine surveillance through staffed shelter kiosks (*19*,*20*). Study enrollment was open to residents >3 months of age reporting new or worsening cough alone or onset of >2 other acute respiratory illness symptoms in the previous 7 days, including subjective fever, sore throat, rhinorrhea, shortness of breath, headache, and myalgias. Symptom criteria also included diarrhea, rash, and ear pain or discharge for children <18 years of age. Persons who did not meet the symptom requirements were allowed to enroll and submit a nasal swab sample while asymptomatic up to once a month for shelter surveillance (i.e., inclusion criteria were broadened to allow a person to participate >1 time per month even if asymptomatic). Beginning April 1, 2020, eligibility expanded to all residents and staff regardless of symptoms as a result of the SARS-CoV-2 response (*19*). Nine shelters participated in the study, which included both participant and environmental testing, before the COVID-19 pandemic (October 2019–March 2020). An additional 14 shelters joined the study during April 2020–May 2021 but only for participant testing because of the need to shift resources toward identification and isolation of persons with SARS-CoV-2 infection.

We obtained written consent from participants >18 years of age or from a guardian for children <18 years of age; we obtained assent from participants 13–17 years of age. We offered $5 gift cards to compensate participants for their time. This study was approved by the Human Subjects Division of the University of Washington Institutional Review Board (approval no. STUDY00007800).

### Data Collection

Study staff recruited participants at each shelter site 3–6 days per week. All participants completed a questionnaire on an electronic tablet and submitted a nasal swab specimen at each enrollment. Questionnaires were stored in Research Electronic Data Capture (https://www.project-redcap.org) and included information on current symptoms, shelter site, and demographics.

We collected respiratory specimens by using midturbinate sterile nylon flocked nasal swabs (FloqSwab; Copan Diagnostics) during October 1, 2019–July 22, 2020, and then subsequently during November 1, 2020–May 31, 2021. During July 22–November 1, 2020, we briefly used anterior nares swabs (US Cotton; SteriPack) because of supply change resource limitations. Given the spread of SARS-CoV-2, we changed the specimen collection protocol to study staff–supervised self-collected swab samples. We shared visual guides with participants before specimen collection to demonstrate self-swabbing.

We collected environmental samples weekly from 9 homeless shelters during November 20, 2019–April 10, 2020. We adapted collection methods described by Bailey et al. (*21*). With residents present, study staff swabbed a 10-cm^2^ area of selected high-touch surfaces (e.g., kitchen counters, front desk, doors, and entrance and restroom doors) by using Berkshire Lab-Tip 125S swabs. We collected bioaerosol samples for 60 minutes in high-traffic areas by using an SKC QuickTake 30 air pump with ambient air pumped through Millipore filter papers. We stored all collected samples in Universal Transport Medium (Copan Diagnostics) and transported on ice.

### Multiplex PCR Testing

We tested nasal swab and environmental samples by using a multiplex reverse transcription PCR platform (Open Array; ThermoFisher Scientific) for 28 viral respiratory pathogen targets, including pan-enterovirus, EV-D68, rhinovirus, influenza viruses (A, B, C), respiratory syncytial viruses (A and B), human parainfluenza viruses (1–4), human coronaviruses, human bocavirus, human parechovirus, human metapneumovirus, adenovirus, and SARS-CoV-2 (from specimens collected beginning January 1, 2020). We generated a relative cycle threshold (Ct) value for each result.

We identified positive or inconclusive enterovirus swabs by using PCR on either pan-enterovirus (ThermoFisher assay Vi06439631_s1) or EV-D68 (ThermoFisher assay Vi06439669_s1) targets and using a relative Ct value of <28 as provided by the manufacturer. Because the enterovirus probe can produce a false-positive test result on a sample with high rhinovirus amplification, laboratory staff reviewed all swab samples initially positive on enterovirus-specific primers and evaluated them on the basis of the degree of enterovirus amplification, enterovirus relative Ct values, and degree of rhinovirus amplification. Finally, we attempted sequencing on all positive or inconclusive enterovirus swabs identified by PCR to confirm enterovirus positivity and subtype.

### Genomic Sequencing and Analysis

To identify viral species and genotypes present in enterovirus-positive swabs, we performed sequencing with enrichment for respiratory viruses using a commercially available panel of capture probes that covered multiple enteroviruses. We attempted whole-genome sequencing on all specimens and environmental samples that were positive or inconclusive for either the pan-enterovirus or EV-D68 targets. In our process, we converted extracted RNA to double-stranded cDNA, purified by bead cleanup, enzymatically fragmented, end-repaired, amplified, indexed, and purified again by using the QIAseq FX DNA Library Kit (QIAGEN, https://www.qiagen.com). We performed hybridization capture by using the QIAseq xHYB Viral Respiratory Panel (QIAGEN) after pooling libraries by sample relative Ct values. After overnight hybridization with biotinylated probes and subsequent washing to remove unbound fragments, we amplified the enriched libraries and purified them by using bead clean-up. We sequenced the resulting libraries on Illumina NovaSeq 6000 or NextSeq 2000 instruments by using a 2 × 150 read format. We generated consensus genomes by using a custom bioinformatic pipeline described previously (Appendix) (*22*).

We categorized specimens and samples as enterovirus-positive when they were positive or inconclusive by PCR and were sequence-confirmed as coxsackievirus or enterovirus. We considered any other sequence-confirmed viruses as enterovirus-negative and grouped them with swabs identified as other respiratory virus (ORV) –positive through PCR testing. We defined enterovirus unknown as any swabs that were initially identified as positive or inconclusive for pan-enterovirus or EV-D68 through PCR but were unable to be sequenced.

### Computational Analysis

We analyzed demographic, symptom, respiratory virus, and environmental data descriptively by using R version 4.3.2 (The R Project for Statistical Computing). We linked multiple enrollments (i.e., encounters) from the same participant by name, date of birth, and sex, as previously described (*18*). We summarized enterovirus results by shelter type and highlighted shelter outbreaks with >5 enterovirus cases. We determined the frequency of enterovirus detection among shelter participants by dividing the number of sequence-confirmed positive specimens by the total number of participant encounters overall and during viral circulation. We used an intercept-only Poisson regression model fitted using generalized estimating equations to obtain robust SE estimates and 95% CIs, accounting for clustering by shelter site. We used NextStrain software to process consensus genomes and to generate and visualize phylogenetic trees (*23*). We calculated bootstrap values using IQ-TREE version 1.6.12 (*24*). In addition to the consensus genomes generated for this study (Appendix Table 1), we downloaded and included in our analyses full-length CVA21 and EV-D68 genomes available from GenBank.

## Results

### Participant Surveillance

**Figure 1:**
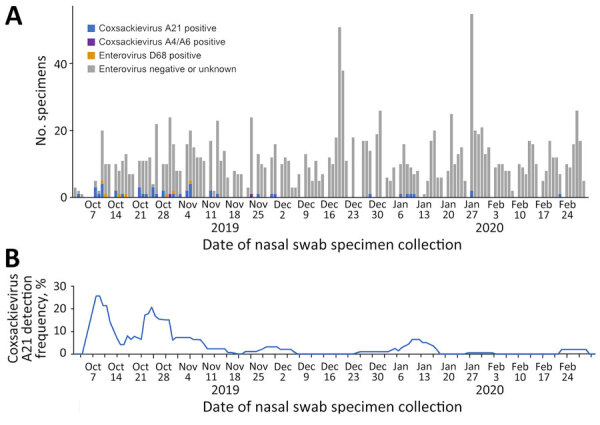
Nasal swab specimens (A) and enterovirus detection (B) in homeless shelters, King County, Washington, USA, October 2019–February 2020. Detection frequency represents a 7-day rolling average. No coxsackievirus A21-positive or enterovirus D68-positive specimens were detected during March 2020–May 2021.

During October 1, 2019–May 31, 2021, we collected 14,464 nasal swab specimens from 3,281 unique participants (22% staff, 78% residents) across 23 homeless shelters (Appendix Table 2, Figure 1). Swabs from children <18 years of age constituted 14% of all specimens collected.

**Figure 2:**
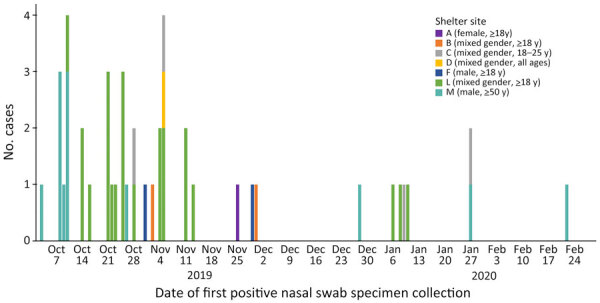
Unique participants with coxsackievirus A21 infection, by homeless shelter site, King County, Washington, USA, October 2019–February 2020.

PCR testing identified 83 participant specimens on either the pan-enterovirus (n = 73) or EV-D68 (n = 46) PCR targets. Upon sequencing, we found 55 confirmed enterovirus-positive specimens among 47 symptomatic shelter residents during October 3, 2019–March 6, 2020 (Figures 1, 2; Appendix Tables 2–4). We detected no enterovirus-positive specimens among shelter staff eligible to participate during April 2020–May 2021. Compared with episodes with enterovirus-negative specimens, episodes with enterovirus-positive specimens were associated with an older median age and being male, being a current tobacco smoker, experiencing chronic homelessness (>1 year), and having underlying conditions (Appendix Table 2). Although the difference in age was attenuated when comparing specimens restricted to enrollment during October 2019–March 2020, other differences remained even after the expansion of eligibility during April 2020–May 2021 (Appendix Tables 3, 4).

We identified cases of CVA21 (n = 39) and EV-D68 (n = 5) among adults and CVA6 (n = 2) and CVA4 (n = 1) among children. Six residents tested CVA21-positive at 2 different timepoints, with a median of 9 days between positive tests (range 2–26 days). Two EV-D68–positive residents tested positive at 2 different timepoints (median 14 days, range 2–26 days). Four coxsackievirus-positive residents had rhinovirus co-detected.

**Figure 3:**
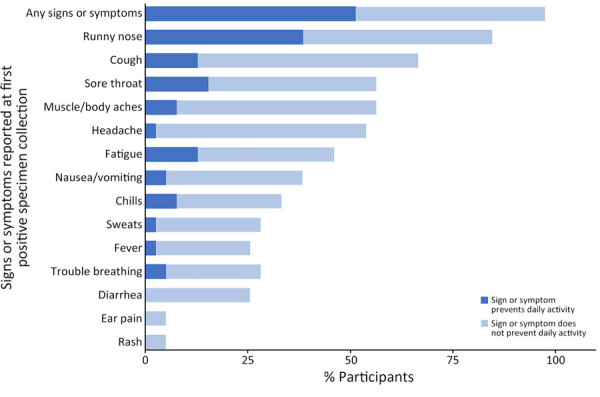
Signs or symptoms reported at specimen collection and effect on daily activity among adult homeless shelter residents with confirmed coxsackievirus A21 infection (n = 39), King County, Washington, USA, October 2019–January 2020. One person with coxsackievirus A21 infection was presymptomatic on initial encounter (first positive specimen collection) but symptomatic on subsequent encounter (second positive specimen collection).

The median age of CVA21-positive persons was 47 years (range 23–72 years). Most (90%) were male; 41% identified as White and 21% as Black/African-American (Appendix Table 2). The most commonly reported signs or symptoms of CVA21 infection included runny nose (85%) and cough (67%) (Figure 3; Appendix Tables 2, 5). Among the 39 unique persons with CVA21 infections, 51% (n = 20) reported a symptom or symptoms that prevented daily activity (Figure 3; Appendix Tables 5, 6). Half of the persons with CVA21 or EV-D68 indicated that their illness affected socialization, followed by those indicating that their illness affected their ability to take care of themselves or their family (36%), exercise (32%), and work (30%). Although 4 CVA21-positive persons sought care at a doctor’s office or an urgent care setting, most (69% of persons with CVA21, 80% of persons with EV-D68) did not seek any medical care (Appendix Table 6).

Overall, CVA21 detection among all participant encounters was 0.3% (45/14,464 [95% CI 0.2%–0.5%]) during October 2019–May 2021 and 3.0% (45/1,485 [95% CI 1.9%–4.8%]) during viral circulation during October 2019–February 2020 (Figure 1; Appendix Table 7). Although we detected CVA21 across 7 shelter sites (Figure 1; Appendix Table 7), most cases occurred in outbreaks at 2 large adult shelters: 19 at mixed-gender shelter L with adults >18 years of age (October 10, 2019–January 10, 2020) (Figure 2; Video 1) and 10 at all-male shelter M with older adults >50 years of age (October 3, 2019–January 27, 2019) (Figure 2; Video 2).

**Video 1:** Maps of confirmed enterovirus cases detected among homeless shelter residents and in environmental samples at shelter L, King County, Washington, USA, October 10, 2019–January 10, 2020. A total of 20 enterovirus-confirmed cases were identified (19 coxsackievirus A21, 1 enterovirus D68); 2 top bunks had multiple residents who were positive for coxsackievirus A21 over time (1 bunk with a resident CVA21-positive on October 21, 2019, followed by a second resident CVA21-positive in the same bunk on November 4, 2019; separately, another bunk had 3 different residents CVA21-positive on 3 dates: October 14, 2019, January 8, 2020, and January, 10, 2020); 1 resident positive for coxsackievirus A21 missing sleeping location; 4 enterovirus-positive environmental samples. Breaks in green lines indicate doorways or entranceways. All dorms have bunk beds (i.e., men’s dormitory consists of 61 bunks, 122 beds total); 2 smaller women’s dormitories are located on another floor. C.R.P., Crisis Response Program.

**Video 2:** Maps of confirmed enterovirus cases detected among homeless shelter residents and in environmental samples at shelter M, King County, Washington, USA, October 3, 2019–January 27, 2020. A total of 14 enterovirus-confirmed cases were identified (10 coxsackievirus A21, 4 enterovirus D68); 3 residents positive for coxsackievirus A21 missing sleeping location; no enterovirus-positive environmental samples. Breaks in green lines indicate doorways or entranceways. Mat placements were ≈9 inches apart in back and front sleeping halls. Site has 2 ultraviolet light air filters located above administrative offices in the front and above men’s lockers in the back.

### Environmental Surveillance

Of 812 environmental swabs, we identified 18 on the pan-enterovirus (n = 8) or EV-D68 (n = 17) PCR targets, and we sequence-confirmed 6 as CVA21 (n = 5) or EV-D68 (n = 1) (Appendix Tables 8, 9, Figure 2). Detection of enterovirus-positive environmental swabs occurred during November 20, 2019–March 12, 2020, across 3 shelters, all which also had resident cases detected. Most CVA21-positive environmental samples (n = 3) were detected at shelter L, which had the largest outbreak of cases among residents (Video 1). Despite having 10 unique CVA21-positive cases and 4 EV-D68-positive cases among its residents, the older adult male shelter (M) did not have any environmental samples that tested enterovirus-positive (Video 2). Surfaces where CVA21 was detected included bathroom doors and the front desk. We detected only 1 sequence-confirmed EV-D68–positive environmental sample from a bathroom door. We detected other viruses in environmental samples through PCR targets more frequently than enteroviruses; the highest rate of detection was for rhinovirus on children’s playroom table (36%, n = 10), front desk (25%, n = 23), and restroom doors (23%, n = 31) (Appendix Table 9). Environmental surfaces tested consisted of plastic, Formica, or metal (Appendix Figure 3). None of the 99 bioaerosol samples tested were positive for enterovirus or another respiratory virus (Appendix Table 9).

### Genomic Analysis

**Figure 4:**
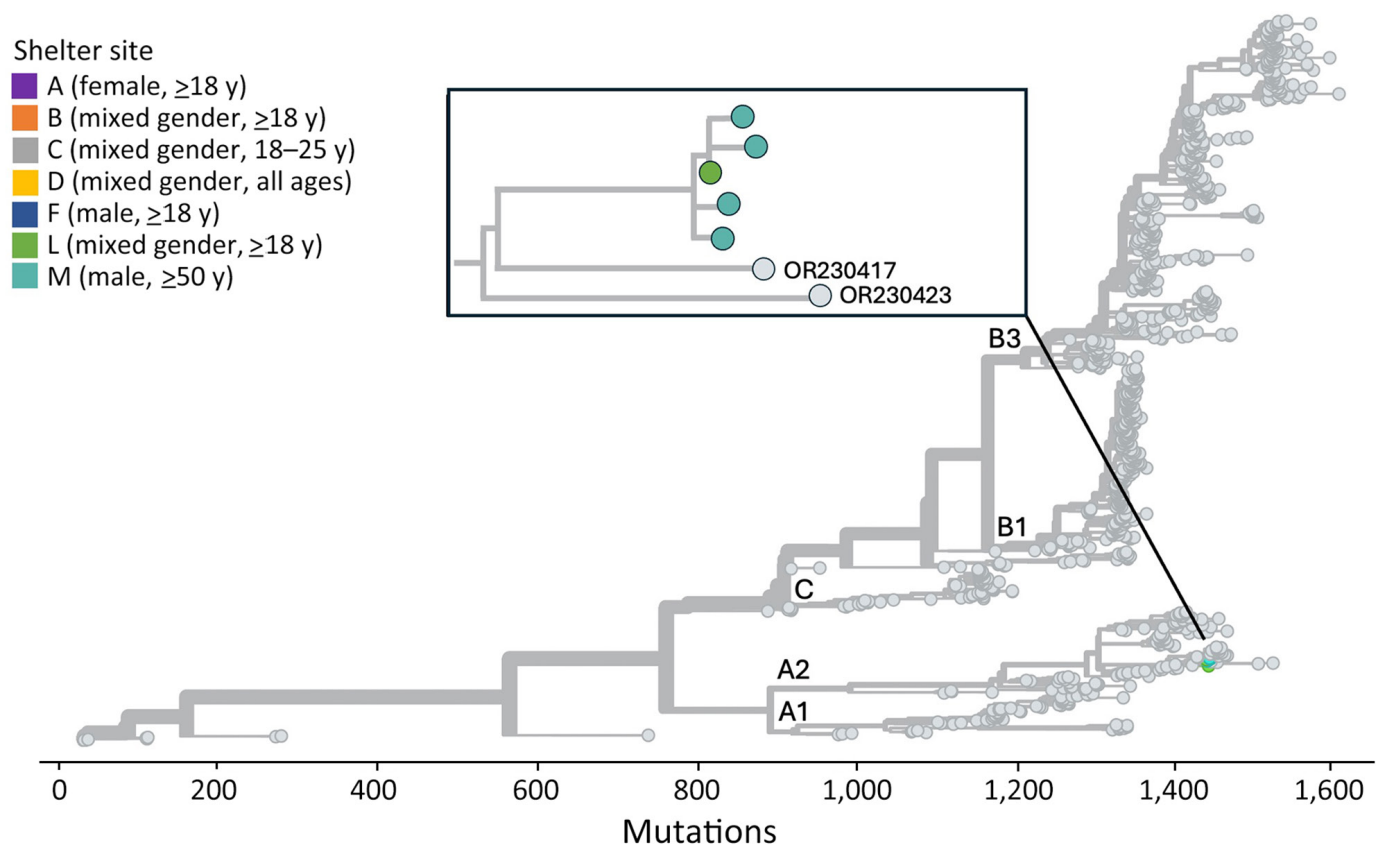
Phylogenetic tree of sequenced enterovirus D68 specimens of homeless shelter residents, King County, Washington, USA, October 2019–November 2019. Tips representing study specimens are colored according to shelter. Light gray tips represent enterovirus D68 genomes downloaded from GenBank. Inset shows a detailed view of the relationship among the study genomes. The x-axis represents the number of nucleotide changes in the genome relative to an enterovirus D68 reference genome (GenBank accession no. NC_038308.1).

Because positive environmental samples may represent mixtures of viruses from multiple shelter residents or staff, we focused our genomic analysis on sequenced species from unique participants (Appendix Table 8). We collected all EV-D68 genomes from 5 unique participants during a 3-week period (October 10–31, 2019) from 2 shelters, L (n = 1) and M (n = 4). These formed a single cluster among 1,032 publicly available EV-D68 genomes downloaded from GenBank (Figure 4); specimens from shelter M did not cluster separately from the specimen from shelter L. All 5 genomes were of EV-D68 clade A2 and among the genomes from GenBank were most closely related to 2 genomes (GenBank accession nos. OR230417 and OR230423) collected in the United States in 2020. The environmental EV-D68 sample also was clade A2 but did not cluster with the participant specimens among the GenBank genomes (Appendix Figure 4).

**Figure 5:**
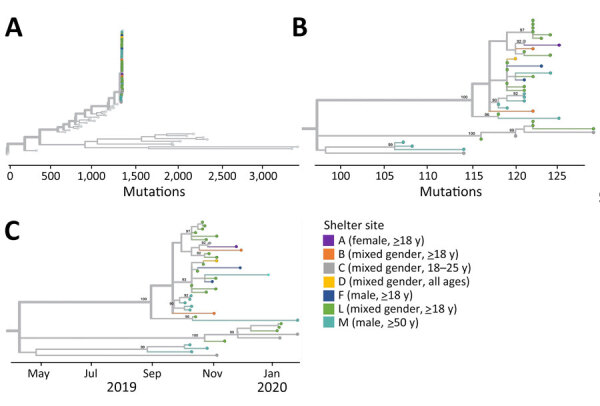
Phylogenetic trees of sequenced coxsackievirus A21 specimens of homeless shelter residents, King County, Washington, USA, October 2019–February 2020. A) Tree containing all shelter coxsackievirus A21 and all coxsackievirus A21 genomes deposited in GenBank. Tips representing study specimens are colored according to shelter. Light gray tips represent coxsackievirus A21 genomes downloaded from GenBank. The x-axis represents number of nucleotide changes in the genome relative to a coxsackievirus A21 reference genome (GenBank accession no. AF465515.1). B) Tree containing all shelter coxsackievirus A21 genomes. Internal nodes with >90% bootstrap support are labeled on tree. C) Tree containing all shelter coxsackievirus A21 genomes with x-axis corresponding to sample collection date.

All CVA21 genomes from 39 unique participants across 7 shelters formed a single phylogenetic cluster among 29 publicly available CVA21 genomes downloaded from GenBank (Figure 5, panel A). The study genomes fall within CVA21 cluster I (*9*,*25*) and are mostly closely related to a genome collected in Nepal in 2017 (GenBank accession no. MZ396299). We observed some clustering by shelter (Figure 5, panels B, C) and instances of identical genomes at the same shelter. The mean pairwise genetic distances between specimens from the same shelter were lower than those from different shelters; however, this difference was not statistically significant (p = 0.0927 by analysis of variance) (Appendix Table 10). We observed no shelters with >2 sequenced participant specimens where all shelter genomes formed a single phylogenetic cluster and, among sequence clusters with >90% bootstrap support, we observed both single and multiple shelter groups. We also noted instances where >1 viral lineage of CVA21 appeared to be circulating at the same shelter at the same time (e.g., shelter M in October 2019). Finally, we observed an association between time of specimen collection and viral genotype given that all 6 specimens collected in 2020 formed a single cluster. Phylogenetic trees including the 5 sequenced environmental CVA21 samples (Appendix Figure 5) illustrate that 4 of 5 environmental samples were closely related to other specimens from the same shelter. The other sample from shelter L was not closely related to any other sequenced shelter specimens and, given its position in the tree, might represent a mixture of viral genotypes observed among the CVA21 shelter specimens.

We visualized the single sequenced CVA4 specimen in a phylogenetic tree among publicly available CVA4 genomes (Appendix Figure 6); the most closely related GenBank genome was collected in Tennessee in April 2015 (GenBank accession no. KY271949). The 2 sequenced CVA6 specimens cluster together among publicly available CVA6 genomes (Appendix Figure 7). The GenBank genome most closely related to these strains was collected in France in 2018 (GenBank accession no. MT814570).

## Discussion

Our study characterizes the epidemiology of enteroviruses among persons experiencing homelessness by using respiratory specimen and environmental surveillance from a community-based shelter setting (*14*). Given the increased risk for infectious disease transmission in congregate shelters and heightened potential for complications because of underlying conditions in many residents, understanding enterovirus epidemiology to prevent and support shelters during outbreaks is important. We detected CVA21 in 3% of all participant specimens tested among King County shelters during October 2019–February 2020, which falls within the range of findings in other global studies (<0.1%–57.0%) (*9*,*26*,*27*). Detection of EV-D68 in the shelters in 2019 is aligned with recent studies in Europe that found upsurges in the 2019 and 2021 seasons (*12*,*28*) compared with the previous biennial pattern observed in even years (e.g., 2014, 2016, 2018, and, to a lesser extent, 2020) (*7*,*29*). We detected no enteroviruses among shelter participants during April 2020–May 2021 despite ongoing surveillance during that period, possibly because stricter COVID-19 pandemic mitigation measures were in place.

All identified CVA21 and EV-D68 infections were in adult shelter residents in adult-only shelters, despite surveillance across children and adults, contributing to the scarce literature available on these viruses in adults (*30*). The manifestations of CVA21 and EV-D68 among symptomatic adult residents were similar and aligned with other adult case-patient reports (*30*,*31*). Half of persons with CVA21 reported a symptom that prevented daily activity; however, most enterovirus-positive persons did not seek any clinical care. Although previous studies have found that children are at higher risk for symptomatic EV-D68 infection than adults (*5*,*32*), we did not identify any positive cases among children in our study despite specimens from children constituting 14% of all specimens collected. In addition, we found no EV-D68–positive environmental surface samples in family shelters; we detected EV-D68–positive and CVA21-positive environmental samples in adult-only shelters.

Environmental monitoring is a minimally invasive method of surveillance for both endemic and emerging respiratory pathogens and could be especially useful as an early indicator of viruses circulating in congregate settings. We found CVA21-positive environmental surface samples across 3 of the 7 shelters with CVA21 detection in nasal swabs. Although we did not find enteroviruses in the bioaerosol samples tested, previous studies have documented aerosol detection in the United States (*33*). We detected CVA21-positive environmental surface samples concurrently with the largest outbreak in adult shelter L, but we did not detect them in the older adult male shelter M outbreak, potentially because of enhanced cleaning procedures including ultraviolet disinfection (shelter M staff, pers. comm., 2020, private meeting). Additional details on shelter disinfection practices were unavailable. Detection of CVA21 most commonly on bathroom doors may be suggestive of a fecal–oral route of transmission, as is observed with many enteroviruses (*2*,*34*). Although CVA21 was detected in nasal swab samples before the positive environmental samples in 3 shelters, this finding probably is reflective of the earlier start of human specimen collection (October 2019) compared with environmental sampling (November 2020).

Our genomic analysis offers insight into the diversity of enteroviruses circulating in King County and the relationships among viruses of the same species within individual shelters and among different shelters. For EV-D68 and CVA21, the study specimens were closely related relative to the diversity represented by publicly available genomes of the same species. This finding may suggest that only 1 lineage of each of these viruses was circulating in King County during the study period, although other lineages not captured in our swab samples might have been present. Of note, very limited information about CVA21 genomic diversity is available, and the sequences generated by our study more than doubled the number of full genomes available for the virus. 

The relationships among shelter CVA21 and EV-D68 genomes were complex. In some cases, viruses from the same shelter clustered together or were even identical, which is consistent with some intra-shelter viral spread. The phylogenetic analysis also identified instances in which viruses were more closely related to specimens from other shelters rather than the same shelter. This finding could be indicative of inter-shelter spread, although our limited knowledge of how quickly these viruses mutate prevents us from assessing whether this finding could represent direct transmission between shelters. For shelters B, C, L, and M, the phylogenetic tree was suggestive of >1 introduction of CVA21 into each shelter during the study period.

Because environmental samples can constitute mixtures of viruses from >1 person deposited at different times, interpretation of their placement in phylogenetic trees is difficult. We observed that CVA21 environmental samples grouped with other study specimens among the genomes from GenBank; in most cases, CVA21 environmental samples appeared most closely related to a participant specimen from the same shelter. This finding indicates that, despite the potentially complex origins of environmental samples, they can offer some insights into viral genotypes circulating at a location and as a result could be extremely valuable in cases where specimens from persons are unavailable.

This study describes the epidemiology of enteroviruses in congregate homeless shelters by using genetically sequenced surveillance data and associated symptom data. Although most previous studies on CVA21 and EV-D68 among adults are from hospitalization data and focus on case reports, our study provides both surveillance and environmental sampling data from a community setting. 

Limitations of our study include the potential for a nonrepresentative sample because of voluntary participation, a lack of site-specific intervention data (e.g., disinfection practices), and a relatively small case count. In addition, limitations of testing include the sample type used (given that nasopharyngeal swab samples historically are considered the standard), collection type used (given potential differences in quality between specimens that are self-collected versus staff-collected), and small sample size of enterovirus data (given the need to restrict to specimens confirmed through sequencing given the cross-reactivity of assays). Our conclusions also are limited by the study’s cross-sectional nature because we could not follow up with participants about potential long-term complications and care-seeking (e.g., hospital admissions). Further research on longitudinal outcomes of enterovirus-positive participants is needed (*12*,*28*).

Our findings provide information on CVA21 and EV-D68 epidemiology, clinical characteristics, and transmission patterns to guide clinical diagnosis and public health interventions. Further understanding of enteroviruses can be used to develop effective preventative measures and treatment options. Surveillance of enteroviruses in shelters and other congregate settings may be warranted for early detection and implementation of control measures to reduce outbreaks.

Appendix Additional information about clinical and genomic epidemiology of coxsackievirus A21 and enterovirus D68 in homeless shelters, King County, Washington, USA, 2019–2021.
