# Supplementary figures and images for: Clinical and Genomic Epidemiology of Coxsackievirus A21 and Enterovirus D68 in Homeless Shelters, King County, Washington, USA, 2019–2021
Source: Emerg Infect Dis. 2024 Nov;30(11):2250–60. doi: 10.3201/eid3011.240687 (PMC11521184; doi:10.3201/eid3011.240687)

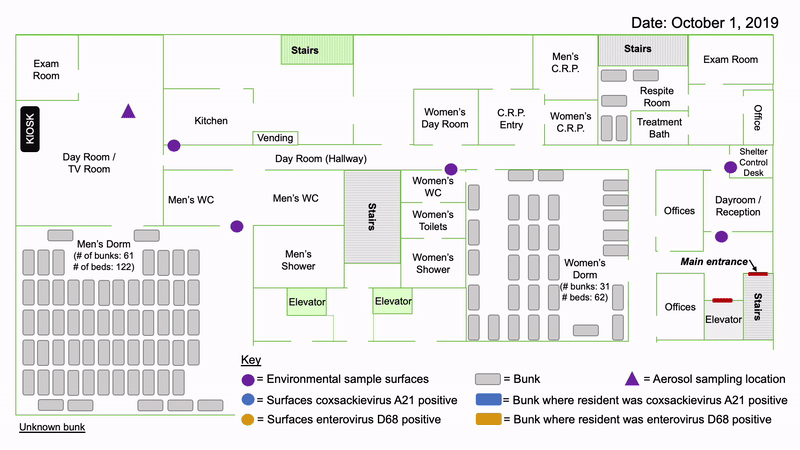

Supplement: Supplementary file 1 [file 24-0687-V1.gif]

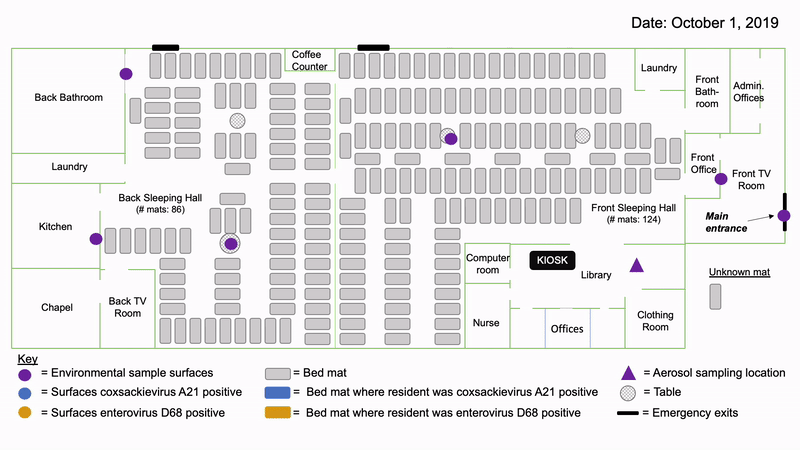

Supplement: Supplementary file 2 [file 24-0687-V2.gif]
