# Supplementary material for: Clinical and Genomic Epidemiology of Coxsackievirus A21 and Enterovirus D68 in Homeless Shelters, King County, Washington, USA, 2019–2021
Source: Emerg Infect Dis. 2024 Nov;30(11):2250–60. doi: 10.3201/eid3011.240687 (PMC11521184; doi:10.3201/eid3011.240687)
Supplement: Appendix — Additional information about clinical and genomic epidemiology of coxsackievirus A21 and enterovirus D68 in homeless shelters, King County, Washington, USA, 2019–2021. [file 24-0687-Techapp-s1.pdf]

Article DOI: https://doi.org/10.3201/eid3011.240687

EID cannot ensure accessibility for supplementary materials supplied by authors. Readers who have difficulty accessing supplementary content should contact the authors for assistance.

Clinical and Genomic Epidemiology of Coxsackievirus A21 and Enterovirus D68 in Homeless Shelters, King County, Washington, USA, 2019–2021

Appendix

Supplemental methods

Data collection and multiplex PCR testing

Respiratory specimens were initially collected using midturbinate sterile nylon flocked swabs (Copan Diagnostics, FLOQSwab 56380CS01) from October 1, 2019-July 22, 2020, and then subsequently from November 1, 2020-May 31, 2021. Anterior nares swabs (SteriPack, U.S. Cotton #3 60564RevB) were used from July 22, 2020-November 1, 2020, due to supply chain resource limitations. Given the spread of SARS-CoV-2, the specimen collection protocol was changed to study staff-supervised self-collected swabs. Comparability of a self-collected mid-turbinate swab to clinician-obtained nasopharyngeal sample has previously been described (*1*,*2*).

All nasal swab specimens and environmental samples were stored at 4°C in universal transport media. Samples were purified for total nucleic acids using the Roche MagnaPure 96 DNA and viral NA small volume kit, Viral NA Universal SV 4·0 protocol (200µL input, 50µL elution) and tested by RT-PCR for multiple viral pathogens using a custom arrayed platform including: enterovirus (pan-enterovirus; EV-D68), rhinovirus, influenza viruses (A, B and C), respiratory syncytial viruses (A&B), human parainfluenza viruses (1–4), human coronaviruses (HCoV), human metapneumovirus, human bocavirus, human parechovirus and adenovirus.

Further details on target strains and cross-reactivity for each assay used are detailed on the ThermoAssay Web site (https://www.thermofisher.com/microbe-detection/taqman/query). In particular, the EV-D68 (Thermo Assay ID: Vi06439669_s1) primer cross-reacts to 16 other taxa

including: Enterovirus C, enterovirus A90, enterovirus A76, enterovirus C96, coxsackievirus A11, coxsackievirus A13, coxsackievirus A19, coxsackievirus A1, coxsackievirus A20, coxsackievirus A22, human enterovirus, coxsackievirus A21, human poliovirus 1, human poliovirus 2, coxsackievirus A24, and enterovirus C99.

Given this known cross-reactivity, all swabs initially positive on the enterovirus-specific primers (Thermo Assay ID: Vi06439631_s1, Vi06439669_s1) were reviewed by laboratory staff using a process recommended by the manufacturer to ensure quality in the differentiation between enterovirus and rhinovirus. Staff evaluated each sample based on the degree of enterovirus amplification, enterovirus Crt values, and the degree of rhinovirus amplification. Swabs initially called enterovirus positive that had low enterovirus amplification ($\Delta RN < 1000$), high enterovirus Crt value (>20), and/or high rhinovirus amplification (Crt<19, $\Delta RN > 1500$) were called enterovirus-negative and the results were adjusted based on this additional review. RN is a measure of the intensity of a marker dye in a PCR mix and the ΔRN refers to the change in RN that occurs when the PCR is run. RN increases when a target is amplified by PCR. A low ΔRN occurs if a target is not present in the PCR mix or if it is present at very low concentrations as the PCR cannot amplify the target sequence without a template. Samples that were true positives for both rhinovirus and enterovirus had high levels of amplifications and low Crt values for both viruses.

Beginning November 23, 2020, the OpenArray platform identified HCoV by species including HCoV-HKU1, HCoV-NL63, HCoV-229E and HCoV- OC43. Beginning February 25, 2020, samples were tested for SARS-CoV-2 in real-time by a multiplexed RT-PCR assay targeting SARS-CoV-2 Orf1b and human RNase P genes in samples collected through March 18, 2020, and a multiplexed RT-PCR assay targeting SARS-CoV-2 Orf1b and S genes with FAM Fluor and the human RNase P gene with VIC or HEX fluor from March 19, 2020, onward. Specimens collected from January 1, 2020-February 24, 2020, were tested retrospectively using a single replicate Orf1b and RNase P multiplexed RT-PCR research assay to detect SARS-CoV-2 Orf1b. An OpenArray relative cycle threshold (Crt) value was calculated for virus-positive samples.

Our custom arrayed RT-PCR panel did not include human bocavirus or human parechovirus during the latter part of the study and may have missed detection of these viruses.

Genomic sequencing and analysis

Briefly, raw reads were trimmed with Trimmomatic (v0.39) using the settings ILLUMINACLIP:2:30:10:1:true, SLIDINGWINDOW: 4:20, LEADING: 3, TRAILING: 3, MINLEN: 35 andmapped to a multi-fasta reference containing complete genomes of multiple respiratory viruses using BBMap (v38.96). The reference with the highest median coverage was selected and trimmed reads were mapped again to the selected reference using BBMap with a strict max indel of 9. The resulting bam was used to call a consensus genome using Samtools (v.1.15) and iVar (v1.3.1) with minimum per-base coverage of 5x, minimum base quality of 20, and minimum frequency threshold of 0.6. Regions with less than the minimum coverage were called Ns. This process was iterated for a total of three times and leading and trailing Ns were trimmed to generate a final consensus.

References

1. McCulloch DJ, Kim AE, Wilcox NC, Logue JK, Greninger AL, Englund JA, et al. Comparison of unsupervised home self-collected midnasal swabs with clinician-collected nasopharyngeal swabs for detection of SARS-CoV-2 infection. JAMA Netw Open. 2020;3:e2016382. PubMed https://doi.org/10.1001/jamanetworkopen.2020.16382

2. Kim AE, Brandstetter E, Wilcox N, Heimonen J, Graham C, Han PD, et al. Evaluating specimen quality and results from a community-wide, home-based respiratory surveillance study. J Clin Microbiol. 2021;59:e02934–20. PubMed https://doi.org/10.1128/JCM.02934-20

Appendix Table 1. Sequence data deposited to NCBI GenBank and SRA (Bioproject PRJNA1029161).*

Strain	Collection Date	Organism	GenBank	BioSample	SRA
RV-C17/USA/WA-UW-087df/2020	2020–11	Rhinovirus C17	OR726586	SAMN37865749	SRR26415519
RV-C17/USA/WA-UW-1a6c3/2020	2020–11	Rhinovirus C17	OR726585	SAMN37865750	SRR26415518
CV-A21/USA/WA-UW-76354/2019	2019–10	Coxsackievirus A21	OR726590	SAMN37865755	SRR26415504
CV-A21/USA/WA-UW-7c271/2019	2019–10	Coxsackievirus A21	OR726589	SAMN37865757	SRR26415502
RV-C1/USA/WA-UW-c9756/2021	2021–01	Rhinovirus C1	OR726587	SAMN37865760	SRR26415516
CV-A21/USA/WA-UW-f0a35/2019	2019–10	Coxsackievirus A21	OR726591	SAMN37865762	SRR26415514
CV-A21/USA/WA-UW-fd6df/2019	2019–10	Coxsackievirus A21	OR726592	SAMN37865766	SRR26415510
RV-C/USA/WA-UW-ff39d/2021	2021–01	Rhinovirus C	OR726588	SAMN37865767	SRR26415509
HAdV-C5/USA/WA-UW-6bd44/2021	2021–01	Human adenovirus 5	OR728260	SAMN37865664	SRR26445869
HPeV-1B/USA/WA-UW-6bd44/2021	2021–01	Human parechovirus 1B	OR728261	SAMN37865665	SRR26445869
PyV/USA/WA-UW-6bd44/2021	2021–01	Polyomavirus sp.	OR728262	SAMN37865666	SRR26445869
CV-A21/USA/WA-UW-6fbc5/2019	2019–10	Coxsackievirus A21	OR833019	SAMN38286755	SRR26856195
CV-A21/USA/WA-UW-2cf8d/2019	2019–10	Coxsackievirus A21	OR833016	SAMN38286756	SRR26856194
CV-A21/USA/WA-UW-26b08/2019	2019–12	Coxsackievirus A21	OR833030	SAMN38286757	SRR26856183
EV-D68/USA/WA-UW-2c8da/2019	2019–10	Enterovirus D68	OR833015	SAMN38286758	SRR26856172
CV-A21/USA/WA-UW-74951/2019	2019–10	Coxsackievirus A21	OR833046	SAMN38286759	SRR26856161
CV-A21/USA/WA-UW-024bb/2020	2020–01	Coxsackievirus A21	OR833029	SAMN38286760	SRR26856150
CV-A21/USA/WA-UW-4c9f1/2019	2019–11	Coxsackievirus A21	OR833018	SAMN38286761	SRR26856145
CV-A21/USA/WA-UW-c93c6/2019	2019–12	Coxsackievirus A21	OR833052	SAMN38286762	SRR26856144
CV-A21/USA/WA-UW-8b43d/2019	2019–10	Coxsackievirus A21	OR833024	SAMN38286763	SRR26856143
CV-A21/USA/WA-UW-b7d06/2019	2019–11	Coxsackievirus A21	OR833049	SAMN38286764	SRR26856142
CV-A21/USA/WA-UW-8014b/2019	2019–10	Coxsackievirus A21	OR833043	SAMN38286765	SRR26856193
CV-A21/USA/WA-UW-60c43/2019	2019–11	Coxsackievirus A21	OR833034	SAMN38286766	SRR26856192
CV-A21/USA/WA-UW-fab63/2019	2019–10	Coxsackievirus A21	OR833059	SAMN38286767	SRR26856191
CV-A21/USA/WA-UW-db519/2019	2019–10	Coxsackievirus A21	OR833054	SAMN38286768	SRR26856190
CV-A21/USA/WA-UW-08763/2020	2020–01	Coxsackievirus A21	OR833044	SAMN38286769	SRR26856189
CV-A21/USA/WA-UW-f5679/2019	2019–11	Coxsackievirus A21	OR833058	SAMN38286770	SRR26856188
EV-D68/USA/WA-UW-0b718/2019	2019–10	Enterovirus D68	OR833014	SAMN38286771	SRR26856187
CV-A21/USA/WA-UW-2965a/2019	2019–11	Coxsackievirus A21	OR833039	SAMN38286772	SRR26856186
CV-A21/USA/WA-UW-9c594/2020	2020–02	Coxsackievirus A21	OR833027	SAMN38286773	SRR26856185
EV-D68/USA/WA-UW-44dd6/2019	2019–11	Enterovirus D68	OR833032	SAMN38286774	SRR26856184
CV-A21/USA/WA-UW-274e8/2019	2019–11	Coxsackievirus A21	OR833036	SAMN38286775	SRR26856182
CV-A21/USA/WA-UW-13009/2020	2020–01	Coxsackievirus A21	OR833045	SAMN38286776	SRR26856181
CV-A21/USA/WA-UW-e0acd/2019	2019–10	Coxsackievirus A21	OR833056	SAMN38286777	SRR26856180
CV-A21/USA/WA-UW-7d205/2019	2019–11	Coxsackievirus A21	OR833021	SAMN38286778	SRR26856179
CV-A21/USA/WA-UW-085c7/2019	2019–10	Coxsackievirus A21	OR833035	SAMN38286779	SRR26856178
CV-A21/USA/WA-UW-50f62/2020	2020–01	Coxsackievirus A21	OR833033	SAMN38286780	SRR26856177
EV-D68/USA/WA-UW-dea74/2019	2019–10	Enterovirus D68	OR833055	SAMN38286781	SRR26856176
EV-D68/USA/WA-UW-b4a24/2019	2019–10	Enterovirus D68	OR833048	SAMN38286782	SRR26856175
CV-A21/USA/WA-UW-517e0/2019	2019–10	Coxsackievirus A21	OR833037	SAMN38286783	SRR26856174
CV-A21/USA/WA-UW-5495c/2019	2019–10	Coxsackievirus A21	OR833042	SAMN38286784	SRR26856173
CV-A21/USA/WA-UW-bc435/2019	2019–11	Coxsackievirus A21	OR833051	SAMN38286787	SRR26856169
CV-A21/USA/WA-UW-018ec/2019	2019–10	Coxsackievirus A21	OR833028	SAMN38286788	SRR26856168
CV-A21/USA/WA-UW-9a01e/2019	2019–10	Coxsackievirus A21	OR833026	SAMN38286789	SRR26856167
CV-A21/USA/WA-UW-feed7/2019	2019–11	Coxsackievirus A21	OR833060	SAMN38286790	SRR26856166
CV-A21/USA/WA-UW-b9e2a/2019	2019–11	Coxsackievirus A21	OR833050	SAMN38286791	SRR26856165
EV-D68/USA/WA-UW-08a64/2019	2019–10	Enterovirus D68	OR833023	SAMN38286792	SRR26856164
CV-A21/USA/WA-UW-8c83c/2019	2019–11	Coxsackievirus A21	OR833025	SAMN38286793	SRR26856163

Strain	Collection Date	Organism	GenBank	BioSample	SRA
HCoV-NL63/USA/WA-UW-15b1d/2019	2019–12	Human coronavirus NL63	OR833061	SAMN38286794	SRR26856162
CV-A21/USA/WA-UW-e5632/2019	2019–10	Coxsackievirus A21	OR833057	SAMN38286795	SRR26856160
CV-A21/USA/WA-UW-5072b/2019	2019–11	Coxsackievirus A21	OR833041	SAMN38286796	SRR26856159
CV-A21/USA/WA-UW-7b0c5/2019	2019–10	Coxsackievirus A21	OR833020	SAMN38286797	SRR26856158
CV-A21/USA/WA-UW-3048d/2019	2019–11	Coxsackievirus A21	OR833040	SAMN38286798	SRR26856157
CV-A21/USA/WA-UW-d0d44/2019	2019–10	Coxsackievirus A21	OR833053	SAMN38286800	SRR26856155
CV-A21/USA/WA-UW-34db8/2019	2019–10	Coxsackievirus A21	OR833031	SAMN38286801	SRR26856154
CV-A21/USA/WA-UW-a04b8/2019	2019–10	Coxsackievirus A21	OR833047	SAMN38286802	SRR26856153
EV-D68/USA/WA-UW-a0771/2019	2019–10	Enterovirus D68	PP025331	SAMN38286803	SRR26856152
CV-A21/USA/WA-UW-706a1/2019	2019–11	Coxsackievirus A21	OR833038	SAMN38286804	SRR26856151
CV-A21/USA/WA-UW-7dfa3/2019	2019–11	Coxsackievirus A21	OR833022	SAMN38286805	SRR26856149
CV-A21/USA/WA-UW-2dbc6/2019	2019–11	Coxsackievirus A21	OR833017	SAMN38286808	SRR26856146

*18 of the 76 sequenced swabs were not submitted to GenBank because their consensus genome was either too short, contained high percentage of the ambiguous base N, or had assembly/annotation issues.

Appendix Table 2. Demographic and clinical characteristics among participants with specimens, October 2019 - May 2021*

Characteristics	Enterovirus positive specimens		Enterovirus negative specimens		Specimens overall (n = 14,446)§
	Coxsackievirus (n = 48)†	Enterovirus D68 (n = 7)	ORV‡ Positive (n = 1,373)	ORV‡ Negative (n = 13,018)	
Age (years), Median [Min, Max]	46 [1, 72]	53 [37, 58]	28 [0, 85]	40 [0, 97]	39 [0, 97]
Age group (years)					
<18	3 (6.3%)	0 (0.0%)	439 (32.0%)	1,594 (12.2%)	2,036 (14.1%)
18–24	3 (6.3%)	0 (0.0%)	159 (11.6%)	1,404 (10.8%)	1,566 (10.8%)
25–49	21 (43.8%)	1 (14.3%)	455 (33.2%)	5,663 (43.5%)	6.140 (42.5%)
50–64	18 (37.5%)	6 (85.7%)	266 (19.4%)	3,503 (26.9%)	3,793 (26.3%)
65+	3 (6.3%)	0 (0.0%)	52 (3.8%)	853 (6.6%)	908 (6.3%)
Sex (biologic)					
Male	42 (87.5%)	7 (100.0%)	802 (58.4%)	7,667 (58.9%)	8,518 (59.0%)
Female	5 (10.4%)	0 (0.0%)	552 (40.2%)	5,176 (39.8%)	5,733 (39.7%)
Other	0 (0.0%)	0 (0.0%)	6 (0.4%)	49 (0.4%)	55 (0.4%)
Prefer not to say	1 (2.1%)	0 (0.0%)	13 (0.9%)	124 (1.0%)	138 (1.0%)
Pregnant	0 (0.0%)	0 (0.0%)	6 (0.4%)	22 (0.2%)	28 (0.2%)
Race					
American Indian/Alaska Native	1 (2.1%)	0 (0.0%)	40 (2.9%)	427 (3.3%)	468 (3.2%)
Asian	0 (0.0%)	0 (0.0%)	14 (1.0%)	401 (3.1%)	415 (2.9%)
Black or African American	8 (16.7%)	5 (71.4%)	402 (29.3%)	4,189 (32.2%)	4,604 (31.9%)
Native Hawaiian/Other Pacific Islander	1 (2.1%)	0 (0.0%)	115 (8.4%)	473 (3.6%)	589 (4.1%)
White	20 (41.7%)	2 (28.6%)	474 (34.5%)	4,972 (38.3%)	5,468 (37.9%)
Multiracial	7 (14.6%)	0 (0.0%)	127 (9.3%)	941 (7.2%)	1,075 (7.5%)
Other	7 (14.6%)	0 (0.0%)	61 (4.4%)	690 (5.3%)	758 (5.3%)
Prefer not to say	4 (8.3%)	0 (0.0%)	140 (10.2%)	900 (6.9%)	1,044 (7.2%)
Hispanic ethnicity	5 (10.4%)	0 (0.0%)	231 (16.8%)	1,640 (12.6%)	1,875 (13.0%)
Shelter staff	0 (0.0%)	0 (0.0%)	130 (9.5%)	3,029 (23.3%)	3,159 (21.9%)
Employed	10 (20.8%)	3 (42.9%)	208 (15.1%)	2,335 (17.9%)	2.556 (17.7%)
Chronic homelessness¶	31 (64.6%)	4 (57.1%)	347 (25.3%)	3,176 (24.4%)	3,558 (24.6%)
Current tobacco smoker#	34 (70.8%)	5 (71.4%)	506 (36.9%)	5,920 (45.5%)	6,465 (44.8%)
Any comorbidities**	25 (52.1%)	0 (0.0%)	313 (22.8%)	3,639 (28.0%)	3,977 (27.5%)
Asthma	9 (18.8%)	0 (0.0%)	164 (11.9%)	1,651 (12.7%)	1,824 (12.6%)
Cancer	3 (6.3%)	0 (0.0%)	16 (1.2%)	237 (1.8%)	256 (1.8%)
Cardiovascular disease	3 (6.3%)	0 (0.0%)	36 (2.6%)	463 (3.6%)	502 (3.5%)
Chronic obstructive pulmonary disease	7 (14.6%)	0 (0.0%)	70 (5.1%)	684 (5.3%)	761 (5.3%)
Diabetes mellitus	4 (8.3%)	0 (0.0%)	102 (7.4%)	1,145 (8.8%)	1,251 (8.7%)
Hepatic disease	2 (4.2%)	0 (0.0%)	20 (1.5%)	388 (3.0%)	410 (2.8%)
Immunosupression	3 (6.3%)	0 (0.0%)	15 (1.1%)	165 (1.3%)	183 (1.3%)
Neurologic disease	7 (14.6%)	0 (0.0%)	20 (1.5%)	114 (0.9%)	141 (1.0%)
Non-enteroviruses co-detected††,‡‡	4 (8.3%)	0 (0.0%)	1,373 (100.0%)	0 (0.0%)	1,377 (9.5%)
Any symptoms††	47 (97.9%)	7 (100.0%)	465 (33.9%)	2,224 (17.1%)	2,743 (19.0%)
Runny nose	41 (85.4%)	7 (100.0%)	356 (25.9%)	1,292 (9.9%)	1,696 (11.7%)
Cough	33 (68.8%)	7 (100.0%)	313 (22.8%)	1,082 (8.3%)	1,435 (9.9%)
Sore throat	26 (54.2%)	1 (14.3%)	183 (13.3%)	671 (5.2%)	881 (6.1%)
Muscle/body aches	24 (50.0%)	1 (14.3%)	169 (12.3%)	720 (5.5%)	914 (6.3%)
Fatigue	22 (45.8%)	3 (42.9%)	167 (12.2%)	755 (5.8%)	947 (6.6%)
Headache	22 (45.8%)	1 (14.3%)	164 (11.9%)	716 (5.5%)	903 (6.3%)
Nausea/vomiting	20 (41.7%)	1 (14.3%)	127 (9.2%)	507 (3.9%)	655 (4.5%)
Fever/feeling feverish	14 (29.2%)	2 (28.6%)	126 (9.2%)	464 (3.6%)	606 (4.2%)
Sweats	15 (31.3%)	2 (28.6%)	98 (7.1%)	432 (3.3%)	547 (3.8%)
Chills	17 (35.4%)	0 (0.0%)	106 (7.7%)	450 (3.5%)	573 (4.0%)
Trouble breathing	12 (25.0%)	0 (0.0%)	102 (7.4%)	352 (2.7%)	466 (3.2%)
Diarrhea	12 (25.0%)	0 (0.0%)	72 (5.2%)	299 (2.3%)	383 (2.7%)
Ear pain	3 (6.3%)	0 (0.0%)	37 (2.7%)	148 (1.1%)	188 (1.3%)
Rash	3 (6.3%)	0 (0.0%)	25 (1.8%)	126 (1.0%)	154 (1.1%)

*Participants could enroll in the study and have a specimen collected once per week, or additionally if new signs or symptoms developed
†Includes 45 coxsackievirus A21 specimens among adults; 1 coxsackievirus A4 and 2 coxsackievirus A6 cases among symptomatic children <10 y in family shelters
‡ORV = Other respiratory virus
§Excludes 18 enterovirus unknown specimens (enterovirus detected in initial PCR testing but unable to be sequenced)
¶Homeless ≥1 y
#Only asked for participants aged 12 y+
**Any comorbidities include: asthma, cancer, cardiovascular disease, chronic obstructive pulmonary disease, diabetes mellitus, hepatic disease, immunosupression, neurologic disease
††At time of specimen collection
‡‡2 participants with coxsackievirus A21 and rhinovirus co-detected; 1 participant with coxsackievirus A4 and both rhinovirus and RSV-A co-detected; 1 participant with coxsackievirus A6 and rhinovirus co-detected

Appendix Table 3. Demographic and clinical characteristics among participants with specimens, October 2019 - March 2020 (n = 1,699).*

	Enterovirus positive specimens		Enterovirus negative specimens		Specimens
Characteristics	Coxsackievirus (n = 48)†	Enterovirus D68 (n = 7)	ORV‡ Positive (n = 370)	ORV‡ Negative (n = 1,274)	overall (n = 1,699)§
Age (years), Median [Min, Max]	46 [1, 72]	53 [37, 58]	42 [0, 81]	49 [0, 84]	48 [0, 84]
Age group (years)					
<18	3 (6.3%)	0 (0.0%)	60 (16.3%)	70 (5.5%)	133 (7.8%)
18–24	3 (6.3%)	0 (0.0%)	33 (9.0%)	87 (6.8%)	123 (7.3%)
25–49	21 (43.8%)	1 (14.3%)	131 (35.6%)	489 (38.4%)	642 (37.9%)
50–64	18 (37.5%)	6 (85.7%)	123 (33.4%)	559 (43.9%)	706 (41.6%)
65+	3 (6.3%)	0 (0.0%)	21 (5.7%)	68 (5.3%)	92 (5.4%)
Sex (biologic)					
Male	42 (87.5%)	7 (100.0%)	236 (63.8%)	940 (73.8%)	1,225 (72.1%)
Female	5 (10.4%)	0 (0.0%)	131 (35.4%)	329 (25.8%)	465 (27.4%)
Other	0 (0.0%)	0 (0.0%)	2 (0.5%)	2 (0.2%)	4 (0.2%)
Prefer not to say	1 (2.1%)	0 (0.0%)	1 (0.3%)	3 (0.2%)	5 (0.3%)
Pregnant	0 (0.0%)	0 (0.0%)	6 (1.6%)	13 (1.0%)	19 (1.1%)
Race					
American Indian/Alaska Native	1 (2.1%)	0 (0.0%)	8 (2.2%)	54 (4.2%)	63 (3.7%)
Asian	0 (0.0%)	0 (0.0%)	3 (0.8%)	24 (1.9%)	27 (1.6%)
Black or African American	8 (16.7%)	5 (71.4%)	97 (26.2%)	307 (24.1%)	417 (24.5%)
Native Hawaiian/Other Pacific Islander	1 (2.1%)	0 (0.0%)	7 (1.9%)	15 (1.2%)	23 (1.4%)
White	20 (41.7%)	2 (28.6%)	167 (45.1%)	592 (46.5%)	781 (46.0%)
Multiracial	7 (14.6%)	0 (0.0%)	53 (14.3%)	116 (9.1%)	176 (10.4%)
Other	7 (14.6%)	0 (0.0%)	25 (6.8%)	114 (8.9%)	146 (8.6%)
Prefer not to say	4 (8.3%)	0 (0.0%)	10 (2.7%)	52 (4.1%)	66 (3.9%)
Hispanic ethnicity	5 (10.4%)	0 (0.0%)	45 (12.2%)	132 (10.4%)	182 (10.7%)
Shelter staff	0 (0.0%)	0 (0.0%)	0 (0.0%)	0 (0.0%)	0 (0.0%)
Employed	10 (20.8%)	3 (42.9%)	54 (14.6%)	236 (18.5%)	303 (17.8%)
Chronic homelessness¶	31 (64.6%)	4 (57.1%)	132 (35.7%)	578 (45.4%)	745 (43.8%)
Current tobacco smoker#	34 (70.8%)	5 (71.4%)	204 (55.1%)	814 (63.9%)	1057 (62.2%)
Any comorbidities**	25 (52.1%)	0 (0.0%)	140 (37.8%)	548 (43.0%)	713 (42.0%)
Asthma	9 (18.8%)	0 (0.0%)	63 (17.0%)	207 (16.2%)	279 (16.4%)
Cancer	3 (6.3%)	0 (0.0%)	6 (1.6%)	31 (2.4%)	40 (2.4%)
Cardiovascular disease	3 (6.3%)	0 (0.0%)	21 (5.7%)	68 (5.3%)	92 (5.4%)
Chronic obstructive pulmonary disease	7 (14.6%)	0 (0.0%)	41 (11.1%)	160 (12.6%)	208 (12.2%)
Diabetes mellitus	4 (8.3%)	0 (0.0%)	50 (13.5%)	151 (11.9%)	205 (12.1%)
Hepatic disease	2 (4.2%)	0 (0.0%)	12 (3.2%)	82 (6.4%)	96 (5.7%)
Immunosupression	3 (6.3%)	0 (0.0%)	8 (2.2%)	29 (2.3%)	40 (2.4%)
Neurologic disease	7 (14.6%)	0 (0.0%)	17 (4.6%)	91 (7.1%)	115 (6.8%)
Non-enteroviruses co-detected††,‡‡	4 (8.3%)	0 (0.0%)	370 (100.0%)	0 (0.0%)	374 (22.0%)
Any symptoms††	47 (97.9%)	7 (100.0%)	340 (91.9%)	1,107 (86.9%)	1,501 (88.3%)
Runny nose	41 (85.4%)	7 (100.0%)	291 (78.6%)	855 (67.1%)	1,194 (70.3%)
Cough	33 (68.8%)	7 (100.0%)	262 (70.8%)	772 (60.6%)	1,074 (63.2%)
Sore throat	26 (54.2%)	1 (14.3%)	150 (40.5%)	437 (34.3%)	614 (36.1%)
Muscle/body aches	24 (50.0%)	1 (14.3%)	151 (40.8%)	540 (42.4%)	716 (42.1%)
Fatigue	22 (45.8%)	3 (42.9%)	153 (41.4%)	515 (40.4%)	693 (40.8%)
Headache	22 (45.8%)	1 (14.3%)	145 (39.2%)	457 (35.9%)	625 (36.8%)
Nausea/vomiting	20 (41.7%)	1 (14.3%)	95 (25.7%)	290 (22.8%)	406 (23.9%)
Fever/feeling feverish	14 (29.2%)	2 (28.6%)	117 (31.6%)	349 (27.4%)	482 (28.4%)
Sweats	15 (31.3%)	2 (28.6%)	90 (24.3%)	322 (25.3%)	429 (25.3%)
Chills	17 (35.4%)	0 (0.0%)	100 (27.0%)	340 (26.7%)	457 (26.9%)
Trouble breathing	12 (25.0%)	0 (0.0%)	88 (23.8%)	239 (18.8%)	339 (20.0%)
Diarrhea	12 (25.0%)	0 (0.0%)	68 (18.4%)	185 (14.5%)	265 (15.6%)
Ear pain	3 (6.3%)	0 (0.0%)	30 (8.1%)	96 (7.5%)	129 (7.6%)
Rash	3 (6.3%)	0 (0.0%)	22 (5.9%)	90 (7.1%)	115 (6.8%)

*Participants could enroll in the study and have a specimen collected once per week, or additionally if new signs or symptoms developed
†Includes 45 coxsackievirus A21 specimens among adults; 1 coxsackievirus A4 and 2 coxsackievirus A6 cases among symptomatic children <10 y in family shelters
‡ORV = Other respiratory virus
§Excludes 18 enterovirus unknown specimens (enterovirus detected in initial PCR testing but unable to be sequenced)
¶Homeless ≥1 y
#Only asked for participants aged 12 y+
**Any comorbidities include: asthma, cancer, cardiovascular disease, chronic obstructive pulmonary disease, diabetes mellitus, hepatic disease, immunosupression, neurologic disease
††At time of specimen collection
‡‡2 participants with coxsackievirus A21 and rhinovirus co-detected; 1 participant with coxsackievirus A4 and both rhinovirus and RSV-A co-detected; 1 participant with coxsackievirus A6 and rhinovirus co-detected

Appendix Table 4. Demographic and clinical characteristics among participants with specimens, April 2020 - May 2021 (n = 12,747).*

Characteristics	Enterovirus negative specimens: ORV[†] Positive (n = 1,003)	Enterovirus negative specimens: ORV[†] Negative (n = 11,744)	Specimens overall (n = 12,747)
Age (years), Median [Min, Max]	24 [0, 85]	39 [0, 97]	38 [0, 97]
Age group (years)			
<18	379 (37.8%)	1,524 (13.0%)	1,903 (14.9%)
18–24	126 (12.6%)	1,317 (11.2%)	1,443 (11.3%)
25–49	324 (32.3%)	5,174 (44.1%)	5,498 (43.1%)
50–64	143 (14.3%)	2,944 (25.1%)	3,087 (24.2%)
65+	31 (3.1%)	785 (6.7%)	816 (6.4%)
Sex (biologic)			
Male	565 (56.4%)	6,727 (57.3%)	7,293 (57.2%)
Female	421 (42.0%)	4,847 (41.3%)	5,268 (41.3%)
Other	4 (0.4%)	47 (0.4%)	51 (0.4%)
Prefer not to say	12 (1.2%)	121 (1.0%)	133 (1.0%)
Pregnant	0 (0.0%)	9 (0.1%)	9 (0.1%)
Race			
American Indian/Alaska Native	32 (3.2%)	373 (3.2%)	405 (3.2%)
Asian	11 (1.1%)	377 (3.2%)	388 (3.0%)
Black or African American	305 (30.4%)	3,882 (33.1%)	4,187 (32.9%)
Native Hawaiian/Other Pacific Islander	108 (10.8%)	458 (3.9%)	566 (4.4%)
White	307 (30.6%)	4,380 (37.4%)	4,687 (36.8%)
Multiracial	74 (7.4%)	825 (7.0%)	899 (7.1%)
Other	36 (3.6%)	576 (4.9%)	612 (4.8%)
Prefer not to say	130 (13.0%)	848 (7.2%)	978 (7.7%)
Hispanic ethnicity	186 (18.5%)	1,508 (12.8%)	1,694 (13.3%)
Shelter staff	130 (13.0%)	3,029 (25.8%)	3,159 (24.8%)
Employed	154 (15.4%)	2,099 (17.9%)	2,253 (17.7%)
Chronic homelessness[‡]	215 (21.4%)	2,598 (22.1%)	2,813 (22.1%)
Current tobacco smoker[¶]	302 (30.1%)	5,106 (43.5%)	5,408 (42.4%)
Any comorbidities[#]	173 (17.2%)	3,091 (26.3%)	3,264 (25.6%)
Asthma	101 (10.1%)	1,444 (12.3%)	1,545 (12.1%)
Cancer	10 (1.0%)	206 (1.8%)	216 (1.7%)
Cardiovascular disease	15 (1.5%)	395 (3.4%)	410 (3.2%)
Chronic obstructive pulmonary disease	29 (2.9%)	524 (4.5%)	553 (4.3%)
Diabetes mellitus	52 (5.2%)	994 (8.5%)	1,046 (8.2%)
Hepatic disease	8 (0.8%)	306 (2.6%)	314 (2.5%)
Immunosupression	7 (0.7%)	136 (1.2%)	143 (1.1%)
Neurologic disease	3 (0.3%)	23 (0.2%)	26 (0.2%)
Non-enteroviruses co-detected**	1,003 (100%)	0 (0.0%)	1,003 (7.9%)
Any symptoms**	125 (12.5%)	1,117 (9.5%)	1,242 (9.7%)
Runny nose	65 (6.5%)	437 (3.7%)	502 (3.9%)
Cough	51 (5.1%)	310 (2.6%)	361 (2.8%)
Sore throat	33 (3.3%)	234 (2.0%)	267 (2.1%)
Muscle/body aches	18 (1.8%)	180 (1.5%)	198 (1.6%)
Fatigue	14 (1.4%)	240 (2.0%)	254 (2.0%)
Headache	19 (1.9%)	259 (2.2%)	278 (2.2%)
Nausea/vomiting	32 (3.2%)	217 (1.8%)	249 (2.0%)
Fever/feeling feverish	9 (0.9%)	115 (1.0%)	124 (1.0%)
Sweats	8 (0.8%)	110 (0.9%)	118 (0.9%)
Chills	6 (0.6%)	110 (0.9%)	116 (0.9%)
Trouble breathing	14 (1.4%)	113 (1.0%)	127 (1.0%)
Diarrhea	4 (0.4%)	114 (1.0%)	118 (0.9%)
Ear pain	7 (0.7%)	52 (0.4%)	59 (0.5%)
Rash	3 (0.3%)	36 (0.3%)	39 (0.3%)

*Participants could enroll in the study and have a specimen collected once per week, or additionally if new signs or symptoms developed
[†]ORV = Other respiratory virus
[‡]Homeless ≥1 y
[¶]Only asked for participants aged 12 y+
[#]Any comorbidities include: asthma, cancer, cardiovascular disease, chronic obstructive pulmonary disease, diabetes mellitus, hepatic disease, immunosupression, neurologic disease
[**]At time of specimen collection

Appendix Table 5. Symptoms and impact on daily activity among shelter residents testing positive for coxsackievirus A21 and enterovirus D68, October 2019 - March 2020, Seattle King County, WA.

Symptom at swab collection	Coxsackievirus A21 positive cases (n = 39)	Symptom impact on daily activity*			Enterovirus D68 positive cases (n = 5)	Symptom impact on daily activity*		
		Mild	Moderate	Severe		Mild	Moderate	Severe
Runny nose	33 (84.6%)	3 (7.7%)	15 (38.5%)	15 (38.5%)	5 (100.0%)	4 (80.0%)	1 (20.0%)	0 (0.0%)
Cough	26 (66.7%)	9 (23.1%)	12 (30.8%)	5 (12.8%)	5 (100.0%)	0 (0.0%)	4 (80.0%)	1 (20.0%)
Sore throat	22 (56.4%)	7 (17.9%)	9 (23.1%)	6 (15.4%)	1 (20.0%)	1 (20.0%)	0 (0.0%)	0 (0.0%)
Muscle/body aches	22 (56.4%)	3 (7.7%)	16 (41.0%)	3 (7.7%)	1 (20.0%)	1 (20.0%)	0 (0.0%)	0 (0.0%)
Fatigue	18 (46.2%)	3 (7.7%)	10 (25.6%)	5 (12.8%)	2 (40.0%)	0 (0.0%)	2 (40.0%)	0 (0.0%)
Headache	21 (53.8%)	7 (17.9%)	13 (33.3%)	1 (2.6%)	1 (20.0%)	1 (20.0%)	0 (0.0%)	0 (0.0%)
Nausea/vomiting	15 (38.5%)	2 (5.1%)	11 (28.2%)	2 (5.1%)	1 (20.0%)	0 (0.0%)	1 (20.0%)	0 (0.0%)
Fever/feeling feverish	10 (25.6%)	4 (10.3%)	5 (12.8%)	1 (2.6%)	2 (40.0%)	1 (20.0%)	1 (20.0%)	0 (0.0%)
Sweats	11 (28.2%)	3 (7.7%)	7 (17.9%)	1 (2.6%)	1 (20.0%)	0 (0.0%)	1 (20.0%)	0 (0.0%)
Chills	13 (33.3%)	4 (10.3%)	6 (15.4%)	3 (7.7%)	0 (0.0%)	0 (0.0%)	0 (0.0%)	0 (0.0%)
Trouble breathing	11 (28.2%)	4 (10.3%)	5 (12.8%)	2 (5.1%)	0 (0.0%)	0 (0.0%)	0 (0.0%)	0 (0.0%)
Diarrhea	10 (25.6%)	5 (12.8%)	5 (12.8%)	0 (0.0%)	0 (0.0%)	0 (0.0%)	0 (0.0%)	0 (0.0%)
Ear pain	2 (5.1%)	2 (5.1%)	0 (0.0%)	0 (0.0%)	0 (0.0%)	0 (0.0%)	0 (0.0%)	0 (0.0%)
Rash	2 (5.1%)	1 (2.6%)	1 (2.6%)	0 (0.0%)	0 (0.0%)	0 (0.0%)	0 (0.0%)	0 (0.0%)
Any symptoms	38 (97.4%)	2 (5.1%)	16 (41.0%)	20 (51.3%)	5 (100.0%)	0 (0.0%)	4 (80.0%)	1 (20.0%)

*Mild = does not interfere with daily activity; Moderate = interferes with daily activity; Severe = prevents daily activity

Appendix Table 6. Demographic and clinical characteristics among unique residents testing positive for coxsackievirus A21 vs. enterovirus D68, October 3, 2019 - January 27, 2020

Characteristics*	Coxsackievirus A21 (n = 39)	Enterovirus D68 (n = 5)	Overall (n = 44) †,‡
Symptom prevents daily activity	20 (51.3%)	1 (20.0%)	21 (47.7%)
Sought care			
Yes - Doctor's office or Urgent Care	4 (10.3%)	0 (0.0%)	4 (9.1%)
Yes - Other	8 (20.5%)	1 (20.0%)	9 (20.5%)
No	27 (69.2%)	4 (80.0%)	31 (70.5%)
Illness impact on			
Ability to take care of self/family	16 (41.0%)	0 (0.0%)	16 (36.4%)
Running errands	11 (28.2%)	2 (40.0%)	13 (29.5%)
Exercise	13 (33.3%)	1 (20.0%)	14 (31.8%)
Looking for work	13 (33.3%)	0 (0.0%)	13 (29.5%)
Work	12 (30.8%)	1 (20.0%)	13 (29.5%)
School	3 (7.7%)	1 (20.0%)	4 (9.1%)
Socializing	19 (48.7%)	3 (60.0%)	22 (50.0%)

*At first positive specimen collection
†1 coxsackievirus A4 and 2 coxsackievirus A6 cases among symptomatic children <10 y in family shelters not shown
‡Excludes 18 enterovirus unknown specimens (enterovirus detected in initial PCR testing but unable to be sequenced)

Appendix Table 7. Coxsackievirus A21 and enterovirus D68 detection frequency among participant specimens overall and during viral circulation*

Virus	%	95% CI
Coxsackvirus A21		
Overall	0.3% (45/14,464)	0.2% - 0.5%
During circulation	3.0% (45/1,485)	1.9% - 4.8%
Enterovirus D68		
Overall	0.0% (7/14,464)	0.0% - 0.1%
During circulation	0.5% (7/1,485)	0.2% - 1.2%

*Overall detection frequency defined as October 2019 – May 2021 (across all data collection); Detection frequency during viral circulation defined as October 2019 – February 2020.

Appendix Table 8. Characteristics of sequenced shelter participant specimens and environmental samples

Category		Enterovirus-positive: Enterovirus D68	Enterovirus-positive: Coxsackievirus A4	Coxsackievirus A6	Coxsackievirus A21	Enterovirus-negative	Enterovirus unknown	Total sequenced*
Total		8	1	2	50	12	28	101
Swab Type	Participant	7	1	2	45	10†	18	83
	Unique Participant	5	1	2	39		18	65
	Environmental	1			5	2‡	10	18
Shelter	A (female, ≥18 y)				1			1
	B (mixed gender, ≥18 y)				3		4	7
	C (mixed gender, 18–25 y)				5	3		8
	D (mixed gender, all ages)		1	1	1	5	7	15
	E (mixed gender, all ages)			1			2	3
	F (male, ≥18 y)				2		2	4
	G (mixed gender, ≥18 y)						1	1
	H (mixed gender, all ages)					2	5	7
	L (mixed gender, ≥18 y)	2			26	2	7	37
	M (male, ≥50 y)	6			12			18

*We generated full genome sequences for 78% (n = 65/83) of participants' nasal swab specimens and 44% (n = 8/18) of environmental swab samples. Since we used an enrichment-based approach targeting multiple respiratory viruses, we also identified non-enteroviruses including rhinovirus A13 and C among participants and human adenovirus E4 and coronavirus HKU1 among environmental samples. Of note, we identified one participant with three co-detected viruses (adenovirus, KI polyomavirus, and parechovirus).
†Includes rhinovirus A13 (n = 2), Rhinovirus C (n = 7), and 1 co-detection of adenovirus 5, KI polyomavirus, and parechovirus 1B (n = 1)
‡Includes human adenovirus E4 (n = 1) and human coronavirus HKU1 (n = 1)

Appendix Table 9. Environmental samples detected via PCR targets by specific location in homeless shelters November 2019 - April 2020, King County, Washington, USA.

Shelter location	Enterovirus	Rhinovirus	Adenovirus	Human bocavirus	Human coronavirus	Human meta-pneumovirus	Human parainfluenza	Influenza	RSV	Total samples collected
Kitchen coffee pot or sugar container handle	2 (7%)	0 (0%)	6 (21%)	0 (0%)	0 (0%)	0 (0%)	1 (3%)	1 (3%)	1 (3%)	29
Electronics- computer keyboards, game controllers	2 (5%)	5 (13%)	8 (21%)	4 (11%)	3 (8%)	1 (3%)	0 (0%)	1 (3%)	1 (3%)	38
Restroom door	6 (4%)	31 (23%)	18 (13%)	10 (7%)	4 (3%)	0 (0%)	5 (4%)	3 (2%)	4 (3%)	137
Table- kid's playroom	1 (4%)	10 (36%)	10 (36%)	6 (21%)	1 (4%)	0 (0%)	1 (4%)	2 (7%)	2 (7%)	28
Table- communal	1 (3%)	3 (9%)	0 (0%)	0 (0%)	0 (0%)	0 (0%)	0 (0%)	0 (0%)	0 (0%)	32
Entry point- front desk	3 (3%)	23 (25%)	20 (22%)	13 (14%)	0 (0%)	0 (0%)	3 (3%)	1 (1%)	2 (2%)	93
Kitchen fridge or microwave handle	1 (2%)	5 (9%)	10 (18%)	6 (11%)	1 (2%)	0 (0%)	2 (4%)	1 (2%)	1 (2%)	55
Kitchen counter	1 (1%)	9 (10%)	9 (10%)	5 (5%)	0 (0%)	0 (0%)	1 (1%)	0 (0%)	0 (0%)	91
Entry point- main entrance door handle	1 (1%)	7 (9%)	18 (23%)	9 (12%)	5 (6%)	1 (1%)	2 (3%)	4 (5%)	1 (1%)	78
Water cooler or fountain	0 (0%)	8 (10%)	5 (6%)	10 (7%)	1 (1%)	0 (0%)	2 (2%)	5 (6%)	2 (2%)	83
Entry point- clinic door handle	1 (0%)	1 (2%)	4 (8%)	2 (4%)	0 (0%)	0 (0%)	0 (0%)	0 (0%)	0 (0%)	49
Air	2 (0%)	0 (0%)	1 (1%)	1 (1%)	1 (1%)	0 (0%)	0 (0%)	0 (0%)	0 (0%)	99
Total	18 (2%)	105 (13%)	109 (13%)	62 (8%)	16 (2%)	0 (0%)	17 (2%)	18 (2%)	14 (2%)	812

Appendix Table 10. Comparison of pairwise genetic distances for same shelter and different shelter genome pairs by ANOVA among participants positive for coxsackievirus A21.

Data Summary				
Groups	N	Mean	Standard deviation	Standard error
Same shelter	224	21.094	18.763	1.254
Different shelter	517	23.501	17.476	0.769

ANOVA Summary					
Source	Degrees of freedom	Sum of squares	Mean square	F-statistic	P-value
Between groups	1	905.617	905.617	2.835	0.093
Within groups	739	236,098.338	319.484		
Total	740	237,003.955			

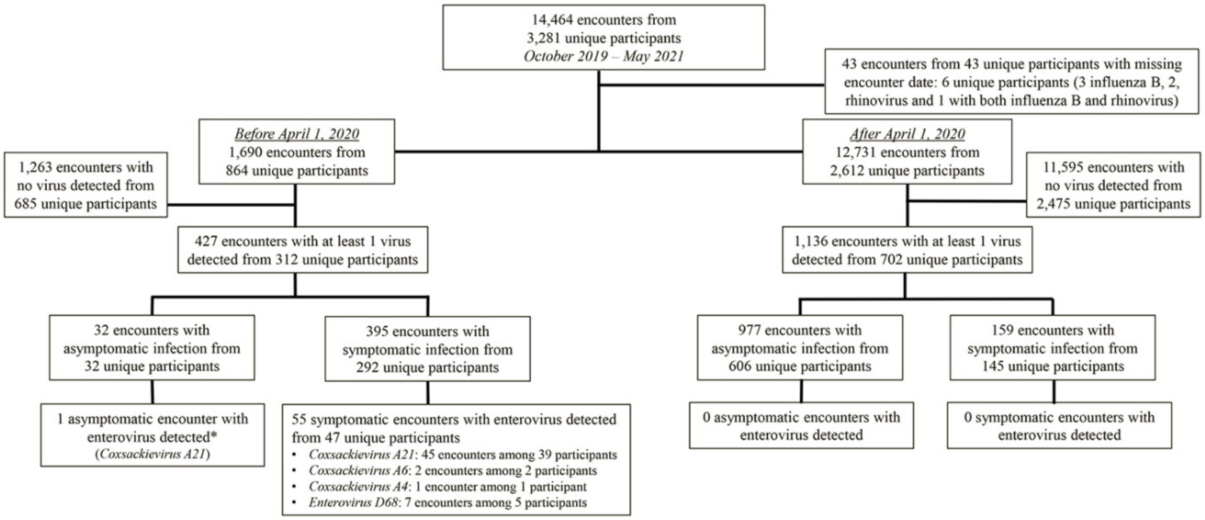

Appendix Figure 1. Homeless shelter study flowchart for enterovirus, King County, Washington, USA.*
*1 asymptomatic enterovirus encounter was among participant that was pre-symptomatic on their initial encounter, but symptomatic on subsequent encounter (included in 39 symptomatic individuals with coxsackievirus A21)

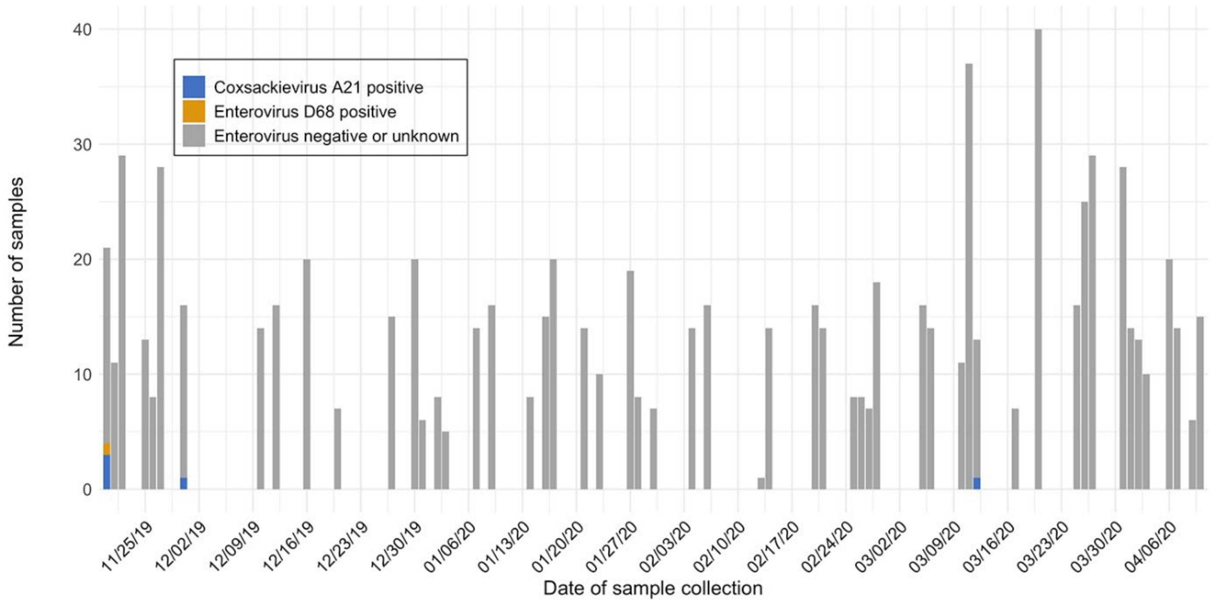

Appendix Figure 2. Environmental samples and enterovirus detection in homeless shelters, November 2019 - April 2020, King County, Washington, USA.* *Environmental samples included high-touch surfaces (i.e., kitchen counters, front desk, doors, and entrance and restroom doors) and bioaerosol samples. See additional details in the main text methods.

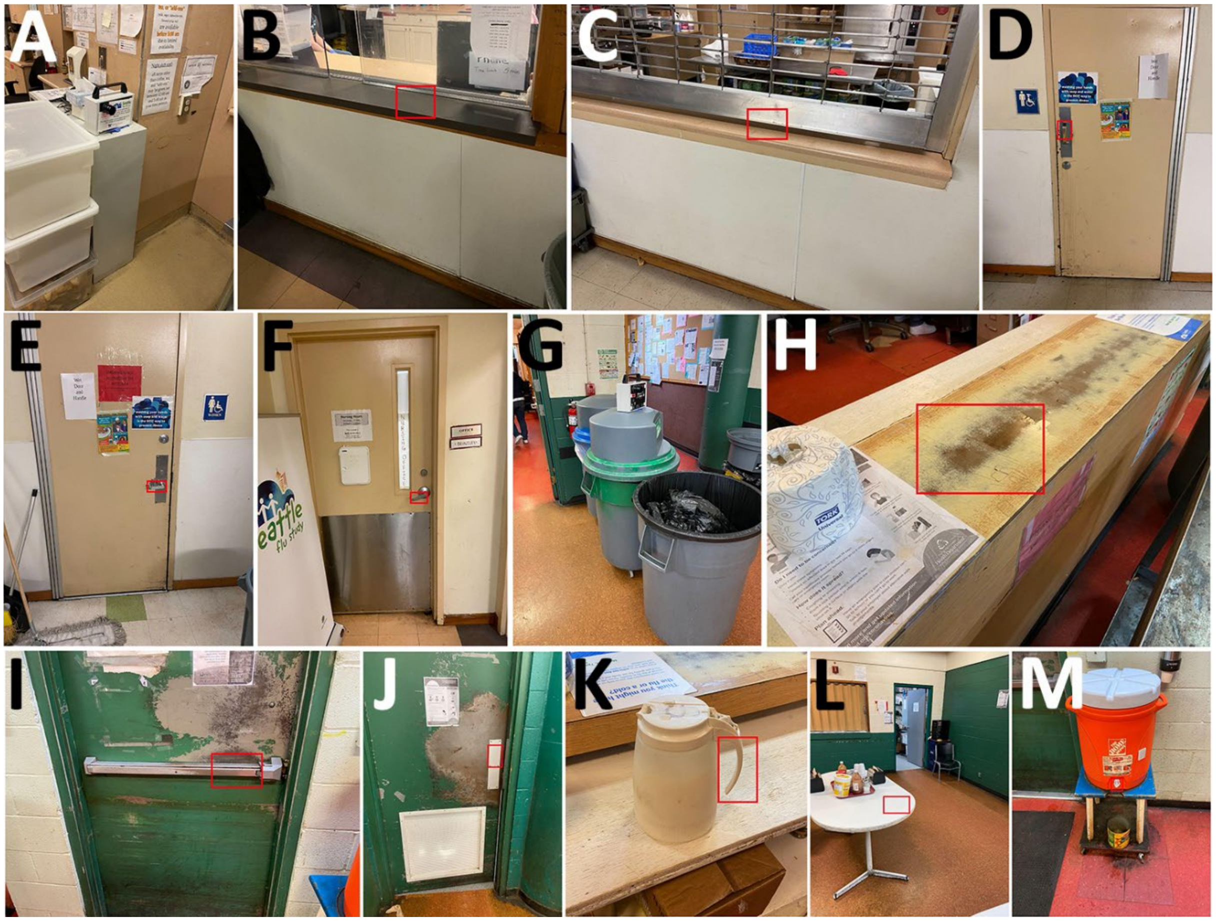

Appendix Figure 3. Photos of environmental sampling locations and surfaces. a. Shelter L air pump. b. Shelter L Front desk. c. Shelter L kitchen counter. d. Shelter L men's bathroom door handle. e. Shelter L women's bathroom door handle. f. Shelter L nurse's room door handle. g. Shelter M air pump. h. Shelter M front desk. i. Shelter M front door handle. j. Shelter M men's bathroom door. k. Shelter M kitchen sugar container handle. l. Shelter M table near kitchen. m. Shelter M water cooler button

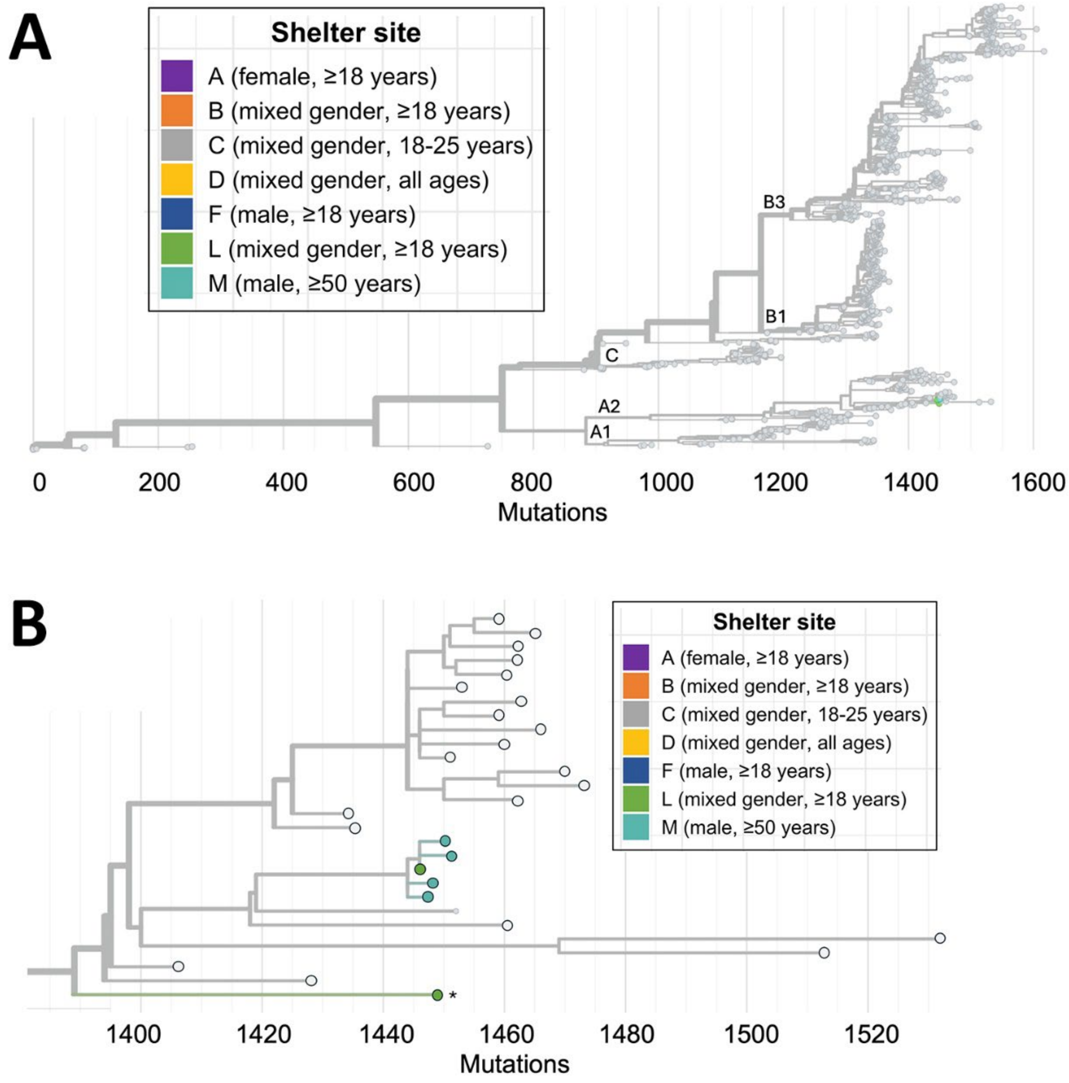

Appendix Figure 4. Phylogenetic trees of all sequenced enterovirus D68 shelter swabs. A) Tree containing all shelter enterovirus D68 and all enterovirus D68 genomes deposited in GenBank. Tips representing study specimens are colored according to shelter. Light gray tips represent enterovirus D68 genomes downloaded from GenBank. The x-axis represents number of nucleotide changes in the genome relative to a enterovirus D68 reference genome (NC_038308.1). B) Tree containing all shelter enterovirus D68 genomes. Environmental sample is labeled with an asterisk.

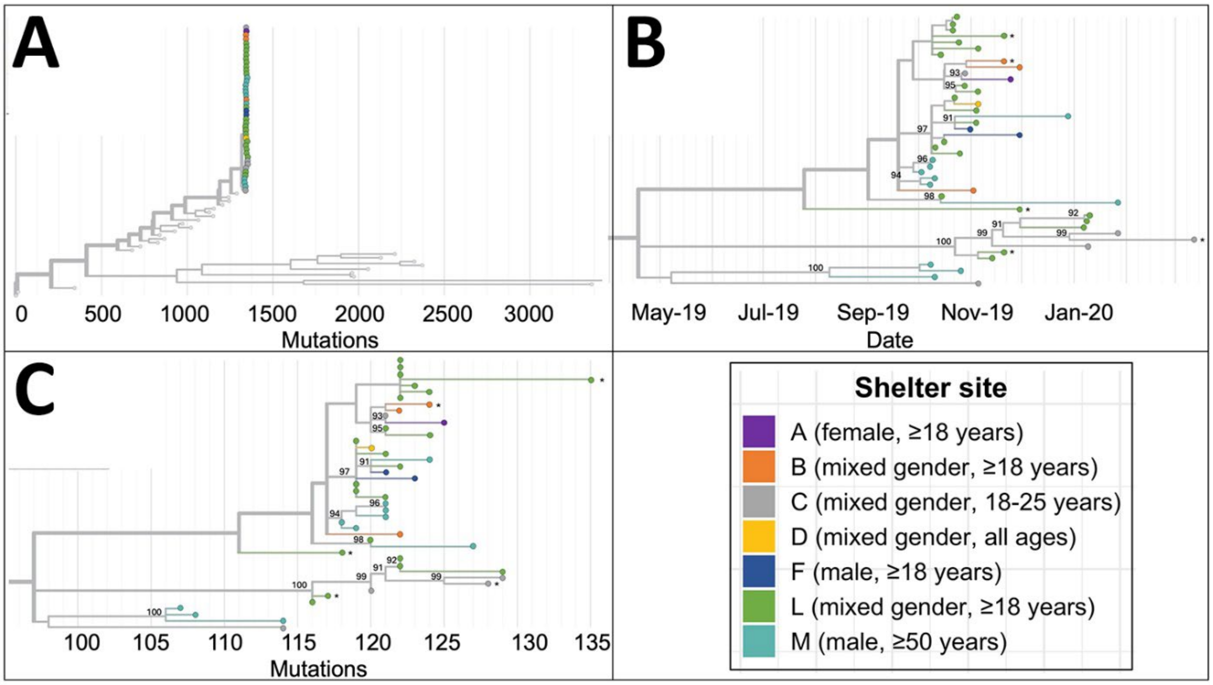

Appendix Figure 5. Phylogenetic trees of all sequenced coxsackievirus A21 shelter swabs. A) Tree containing all shelter coxsackievirus A21 and all coxsackievirus A21 genomes deposited in GenBank. Tips representing study samples are colored according to shelter. Light gray tips represent coxsackievirus A21 genomes downloaded from GenBank. The x-axis represents number of nucleotide changes in the genome relative to a coxsackievirus A21 reference genome (AF465515.1). B) Tree containing all shelter coxsackievirus A21 genomes. Internal nodes with >90% bootstrap support are labeled on tree. Environmental samples are labeled with an asterisk. C) Tree containing all shelter coxsackievirus A21 genomes with x-axis corresponding to sample collection date.

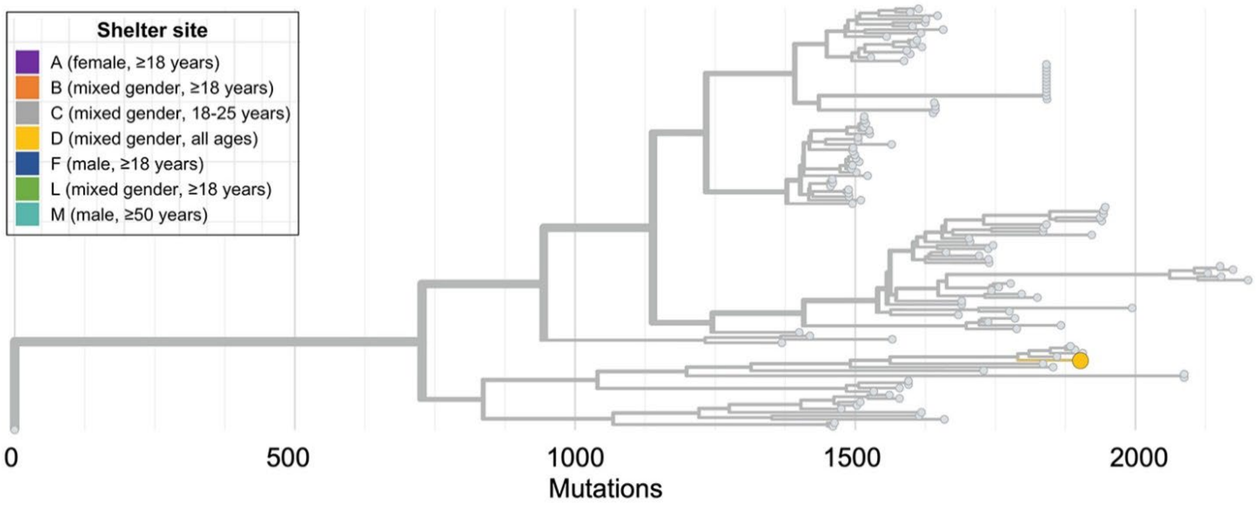

Appendix Figure 6. Phylogenetic tree containing sequenced participant coxsackievirus A4 shelter specimen. The tip representing the study specimen is colored according to its shelter of origin. Light gray tips represent coxsackievirus A4 genomes downloaded from GenBank. The x-axis represents number of nucleotide changes in the genome relative to a coxsackievirus A4 reference genome (AY421762.1).

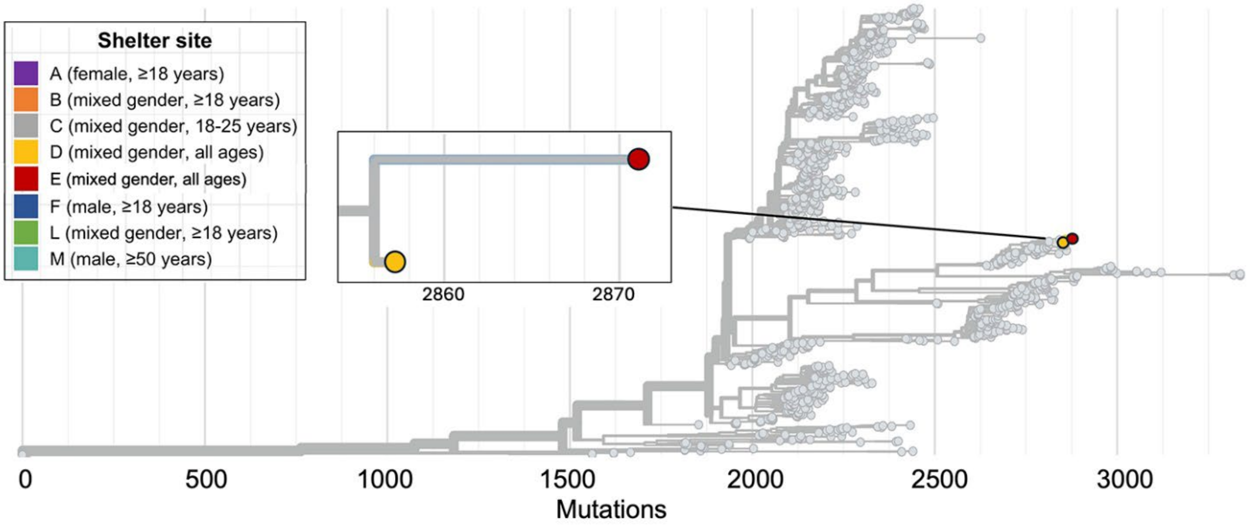

Appendix Figure 7. Phylogenetic tree containing sequenced participant coxsackievirus A6 shelter specimens. The tips representing study specimens are colored according to shelter of origin. Light gray tips represent coxsackievirus A6 genomes downloaded from GenBank. The inset shows a detailed view of the relationship among the study genomes. The x-axis represents number of nucleotide changes in the genome relative to a coxsackievirus A6 reference genome (AY421764.1).
